# Multicenter Analysis of Presacral Neuroendocrine Neoplasms—Clinicopathological Characterization and Treatment Outcomes of a Rare Disease

**DOI:** 10.3389/fendo.2021.709256

**Published:** 2021-10-06

**Authors:** Sami Matrood, Leonidas Apostolidis, Jörg Schrader, Sebastian Krug, Harald Lahner, Annette Ramaswamy, Damiano Librizzi, Zoltan Kender, Anke Kröcher, Simon Kreutzfeldt, Thomas Matthias Gress, Anja Rinke

**Affiliations:** ^1^ Department of Gastroenterology and Endocrinology, UKGM Marburg and Philipps University, Marburg, Germany; ^2^ Department of Medical Oncology, National Center for Tumor Diseases (NCT) Heidelberg, Heidelberg University Hospital, Heidelberg, Germany; ^3^ I. Department of Medicine, University Medical Center Hamburg-Eppendorf, Hamburg, Germany; ^4^ Clinic for Internal Medicine I, Martin-Luther University Halle/Wittenberg, Halle, Germany; ^5^ Department of Endocrinology and Metabolism, University Hospital Essen, Essen, Germany; ^6^ Institute of Pathology, UKGM Marburg and Philipps University, Marburg, Germany; ^7^ Department of Nuclear Medicine, UKGM Marburg and Philipps University, Marburg, Germany; ^8^ Department of Internal Medicine I and Clinical Chemistry, Heidelberg University Hospital, Heidelberg, Germany; ^9^ Clinic for Internal Medicine I, University Hospital Carl Gustav Carus Dresden, Dresden, Germany; ^10^ Department of Translational Medical Oncology, National Center for Tumor Diseases (NCT) Heidelberg and German Cancer Research Center (DKFZ), Heidelberg, Germany

**Keywords:** presacral, retrorectal, CUP-NET, neuroendocrine tumor, neuroendocrine carcinoma, carcinoid, PRRT, prognosis

## Abstract

**Background and Aims:**

Neuroendocrine neoplasms (NENs) of the presacral space are an extremely rare disease entity with largely unknown outcome and no established standard of care treatment. Therefore, we wanted to analyze clinical presentation, histopathological findings, treatment outcomes, and prognosis in a multicentric patient cohort.

**Methods:**

We searched local databases of six German NEN centers for patients with presacral NEN. Retrospective descriptive analyses of age, sex, stage at diagnosis, symptoms, grade, immunohistochemical investigations, biomarkers, treatment, and treatment outcome were performed. Kaplan–Meier analysis was used to determine median overall survival.

**Results:**

We identified 17 patients (11 female, 6 male) with a median age of 50 years (range, 35–66) at diagnosis. Twelve cases presented initially with distant metastases including bone metastases in nine cases. On pathological review the majority of patients had well-differentiated G2 tumors. Immunohistochemical profile resembled rectal NENs. All but one patient had non-functioning tumors. Somatostatin receptor imaging was positive in 14 of 15 investigated cases. Eight patients were treated surgically including palliative resections; 14 patients received somatostatin analogs with limited efficacy. With 14 PRRTs completed, 79% showed clinical benefit, whereas only one patient with neuroendocrine carcinoma (NEC) responded to chemotherapy. Treatment with everolimus in three patients was not successful, whereas cabozantinib resulted in a disease stabilization in a heavily pretreated patient. During a median observation period of 44.5 months, 6 patients died. Median overall survival was not reached.

**Conclusion:**

Presacral NEN are histopathologically similar to rectal NENs. Presacral NEN should be considered as possible primary in NEN of unknown primary. The majority of tumors is non-functioning and somatostatin receptor positive. PRRT demonstrated promising activity; tyrosine kinase inhibitors warrant further investigations. Further molecular characterization and prospective evaluation of this rare tumor entity are needed.

## Introduction

Neuroendocrine neoplasms (NENs) are heterogeneous neoplasms originating from the diffuse neuroendocrine cell system. They are defined by their endocrine phenotype, which is verified by immunohistochemical staining for the small synaptic vesicle-analogue protein synaptophysin and the large dense core-vesicle protein chromogranin A (1). They may originate nearly everywhere in the body, but most often, the primary tumor is located in the gastroenteropancreatic system or in the lung (2). For treatment planning, the knowledge of the primary and the differentiation between primary and metastatic lesion is important. Despite improvement of diagnostic techniques in 8–12% of the NEN patients, the primary remains undetected (CUP-NEN; cancer of unknown primary) (2–5). Somatostatin receptor (SSTR) expression is characteristic for neuroendocrine tumors (NETs) and allows detection of SSTR-expressing NETs by scintigraphy or specific SSTR-PET/CT (68Ga DOTATOC- or DOATATATE-PET-CT) (6). 68Ga-DOTA PET/CTs are particularly important for primary tumor search in CUP-NET, as their sensitivity is superior to other imaging modalities (7).

Specific immunohistochemical stainings including the transcription factors CDX-2 (intestinal primary), TTF-1 (lung/thyroid gland), Islet-1 (pancreas), PDX1 (duodenum, pancreas) (8), and specific hormones may help to identity the primary and are therefore recommended in CUP-NET patients (9). These markers are of very limited use in NEC (9). Prostate-specific acid phosphatase (PSAP) is a glycoprotein-enzyme produced in prostate carcinomas, particularly indicative of its spread beyond the prostate but also characteristic of hindgut NETs (10). In patients with hindgut NETs staining for chromogranin A often is only weakly positive or may even be negative (9, 11).

The presacral space lies between the rectum anteriorly, the sacrum posteriorly, and the endopelvic fascia laterally. It contains embryological remnants of different tissues. Tumors of this presacral space are rare, mostly benign, but several malignant tumors have also been reported (12), including NEN. Immunohistochemistry is important for the differentiation of NEN from other primary tumors or metastases of the presacral region (9, 13). Presacral NENs are extremely rare; to the best of our knowledge, about 70 cases have been reported so far mainly in single case reports (14–70) or small series (15, 17, 20, 26, 39, 42, 55, 71, 72). The majority of presacral NEN was diagnosed in female individuals of younger age compared to the median age of diagnosis in other gastroenteropancreatic NEN. According to the literature, presacral NENs are usually well-differentiated tumors with local involvement, but cases with distant metastases have also been reported (20, 72).

Therapeutic options of metastatic NEN include somatostatin analogs (SSA), chemotherapy, peptide receptor radionuclide therapy (PRRT), everolimus, and tyrosine kinase inhibitors (TKIs) (73). Even in more common NENs like pancreatic NEN, data of comparative treatment trials or on best sequence of treatments are not available at the moment. In rare subtypes like presacral NEN, data on treatment outcome are lacking. The aim of our study is to describe clinical, histopathological, therapeutic, and prognostic features of patients with presacral NEN who presented at one of five contributing NEN referral centers within the last 10 years. We were particularly interested in the number of patients we could collect in the participating NEN referral centers as a surrogate for the frequency of this disease, in the percentage of patients who were initially diagnosed as CUP-NEN, to analyze whether all had differentiated tumors and get a hint which therapeutic option may be of benefit in this extremely rare subgroup.

## Patients and Methods

All patients with neuroendocrine neoplasm and suspected primary tumor within the presacral space were included in this retrospective multicenter evaluation. In the case of initial presentation as NEN with unknown primary, investigations to detect the primary/exclude another primary tumor localization included gastroscopy, colonoscopy, CT or MRI, SSTR imaging [scintigraphy or specific positron emission tomography (PET)] and in some cases fluorodeoxyglucose (FDG)-PET-CT and endoscopic ultrasound. For the vast majority of patients (14 of 17), SSTR-based PET-CT was available during follow-up. In addition to the standard immunohistochemical stainings, such as synaptophysin, chromogranin A, and Ki67, further specific stainings were done according to local practice at the centers, e.g., for the transcriptional factors CDX-2 and TTF-1 in the majority of cases, and ISLET-1, prostate-specific acid phosphatase (PSAP), vimentin, CD56, and somatostatin receptor subtype 2 (SSTR2) in some cases. Several patients received molecular diagnostics *via* “next generation sequencing” panels or whole exome/genome sequencing as part of the German Cancer Consortium (DKTK) Molecularly Aided Stratification for Tumor Eradication Research Trial (MASTER) (74–76).

The patients were identified *via* center-based databases or personal knowledge, and the available essential information was extracted and evaluated across centers. The following German centers have participated: Dresden, Essen, Halle (Saale), Hamburg, Heidelberg, and Marburg. Collected data included date of diagnosis, date of birth, sex, histology, stage, functionality, symptoms of tumor disease, localization of metastases, date of diagnosis of metastases, somatostatin receptor status, treatments with outcome, and date and cause of death or date of last contact. This study was conducted in accordance with the Declaration of Helsinki. All patients were included in the local disease databases conducted with approval of the local ethics committees at the respective sites. Written informed patient consent and approval for data collection and analysis were obtained upon admission to our institutions. For the use of the images, an additional consent was obtained in the selected cases.

Statistical analysis was performed IBM^®^ SPSS^®^ Statistics 27.0 (IBM, Armonk, NY, USA). Descriptive statistical analysis was performed for most parameters. Kaplan–Meier analyses of median duration of observation and overall survival were investigated.

## Results

### Patient Characteristics and Clinical Presentation

We identified 17 patients (n = 17) in the databases of six German centers for neuroendocrine neoplasms [Dresden, Essen, Hamburg, Halle (Saale), Heidelberg and Marburg], who were diagnosed with a primary presacral NEN. Most patients were referred to one of our centers with the diagnosis of cancer of unknown primary (CUP) NET. Presacral NENs were diagnosed more frequently in women (n = 11; 64.7%) than in men (n = 6; 35.3%). In our study population, the initial diagnosis occurred at an age between 35 and 66 years. The patients had a median age of 50 years (mean, 50.3 years).

**Figure 1:**
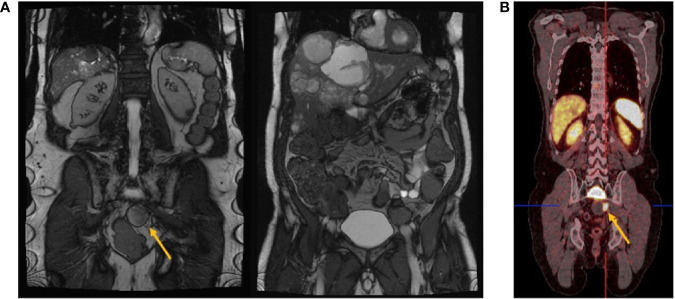
**(A)** Magnetic resonance imaging. Coronal view demonstrating primary presacral neuroendocrine neoplasm (yellow arrow) and liver metastases. **(B)** Coronal view of a 68Ga-DOTATOC-PET/CT scan showing SSTR expression of the whole body. While the liver metastases showed a homogeneous SSTR expression, only a part of the presacral lesion showed a homogeneous SSTR expression (yellow arrow), suggesting a SSTR-negative/cystic portion besides the SSTR-positive solid presacral NEN. MRI and 68-DOTATOC-PET/CT are from the same patient (study-ID III) at different time points.

As mentioned above, an association with various anomalies such as tailgut cysts is frequently described in the literature (72). In our database, an association to an anomaly was detected in only one patient and suspected in another one. One patient showed a presacral localized histologically confirmed teratoma in addition to her primary presacral NEN G2. In another patient, a paraganglioma in the pterygopalatine fossa was suspected but could not be clearly distinguished from osseous metastasis due to a lack of histopathological confirmation. Even though there was no direct association with tailgut cysts, cystic portions of otherwise solid presacral NET could be detected on imaging in some cases (**Figure 1**).

According to our database analysis, presacral NENs are predominantly non-functioning. Only one patient had a functionally active presacral NET producing parathyroid hormone-related peptide (PTHrP). This patient developed a seizure due to paraneoplastic hypercalcemia. No patient suffered from carcinoid syndrome.

Most of the patients (14/17) presented clinically with locoregional symptoms caused by the space-occupying process of presacral NEN. Primary presacral NEN predominantly caused symptoms such as pain of the lower abdomen, pelvis, sacral region, perineum, or lower back (12/17); unilateral paresthesia of the lower limb (2/17); and defecation disorders, e.g., chronic constipation (4/11) or urination disorders (1/17) due to their mass effect. Systemic symptoms showed a minor role in presacral NEN. Only two patients presented with b-symptoms at initial diagnosis.

Patients with presacral NEN were often diagnosed at an advanced stage. At the time of diagnosis, most primary tumors showed a pronounced local extension with a size of 3–9 cm in diameter and frequently an infiltration of the sacrum and the coccyx. In our cohort, most patients (12/17) had distant metastases at the time of diagnosis. Only five patients had a localized tumor stage. However, all patients except one with presacral NEN developed distant metastasis during the course of their disease in our series. Interestingly, one of the most frequent metastatic site in our cohort of patients with presacral NEN was the skeleton. Bone metastases were detected in 11 of 17 patients. Likewise, the liver was a common metastatic site (11/17). Furthermore, locoregional lymph node metastases occurred more frequently (10/17), whereas diffuse lymphatic metastasis to para-aortic and mesenteric lymph nodes and pulmonary metastases occurred less frequently (3/17). Metastases to the adrenal gland and peritoneum were diagnosed in two cases each (2/17). Brain metastases were not diagnosed in our cohort.

Patients characteristics are summarized in **Table 1**.

**Table 1:** Patient characteristics.

Study ID	Sex	Age at diagnosis	Grading	Stage at diagnosis	Endocrine function	SSTR imaging	Symptoms related to presacral NEN	Associated anomalies
*I*	M	48	G2	IV	n.a.	Positive	Perineal pain	–
*II*	F	35	G2	IV	Non-functional	Positive	Defecation disorder	–
*III*	M	65	G2	IV	Non-functional	Positive	Asymptomatic	–
*IV*	F	46	G2	II	Non-functional	Positive	Abdominal and pelvic pain	–
*V*	F	66	G2	IV	Non-functional	Positive	Chronic obstipation	–
*VI*	M	53	G2	IV	Non-functional	Positive	Defecation disorder, perineal pain	–
*VII*	F	52	G3	IV	Non-functional	Positive	Abdominal pain	–
*VIII*	F	40	G3	III	Non-functional	Positive	Asymptomatic	–
*IX*	M	60	G3/LCNEC	III	Non-functional	n.a.	Pain in the sacral region, paresthesia right lower limb, chronic obstipation	–
*X*	F	44	G2	IV	Non-functional	Positive	Pain in the sacral region	–
*XI*	F	65	G2	IV	Non-functional	Positive	Abdominal pain, diffuse backpain	–
*XII*	F	33	G2	IV	Non-functional	Positive	Asymptomatic	–
*XIII*	M	62	G3	III	Non-functional	Positive	Low backpain, paresthesia of the right lower limb, foot drop	–
*XIV*	F	41	G1	III or IV	Non-functional	n.a.	Pelvic pain	Paraganglioma, DD: bone metastasis
*XV*	M	50	G2	IV	Non-functional	Positive	Pelvic pain and swelling of the right hip	–
*XVI*	F	58	G2	IV	Parathyroid hormone-related peptide	n.a.	Pelvic pain, urinary tract obstruction, seizure due to paraneoplastic hypercalcemia	–
*XVII*	F	37	G2	IV	Non-functional	Positive	Pain in the sacral region	Teratoma

n.a., not assessed.

### Histopathological Features

Predominantly, presacral NENs were histologically well differentiated. Most presacral NETs were classified as G2 tumors based on their Ki67 index. Only one presacral NET corresponded to a G1 NET with a Ki67 index of <2%, and three patients had a G3 presacral NET. Poorly differentiated presacral NEC turned out to be extremely rare. In our databases, there was only one presacral NEN that was histopathologically classified as large-cell neuroendocrine carcinoma (LCNEC) and had a Ki67 index of 80%.

Synaptophysin was strongly positive by immunohistochemistry in all samples of presacral NEN. In contrast, chromogranin A was only weakly positive in the majority of cases and even negative in two cases (**Table 2**).

**Table 2:** Immunohistochemical features of patients with primary presacral neuroendocrine neoplasms.

Study ID	Grading	Ki67	Chromogranin A	Synaptophysin	CD56	PSAP	Vimentin	TTF-1	CDX2	CK-7	CK-18
*I*	G2	5%	Negative	Positive	Positive	n.a.	n.a.	n.a.	n.a.	n.a.	n.a.
*II*	G2	12%	Positive	Positive	n.a.	Positive	Negative	Negative	Negative	Negative	n.a.
*III*	G2	7%	Negative	Positive	n.a.	n.a.	Negative	n.a.	Negative	n.a.	n.a.
*IV*	G2	−20%	Weak positive	Positive	n.a.	Positive	n.a.	Negative	Negative	n.a.	n.a.
*V*	G2	10%	Weak positive	Positive	n.a.	Positive	n.a.	Negative	Negative	Negative	Positive
*VI*	G2	5%	Weak positive	Positive	n.a.	Positive	Positive	Negative	Negative	n.a.	n.a.
*VII*	G3	30%	n.a.	Positive	n.a.	n.a.	n.a.	n.a.	n.a.	n.a.	n.a.
*VIII*	G3	30%	Weak positive	Positive	n.a.	n.a.	n.a.	n.a.	n.a.	Negative	Positive
*IX*	G3/LCNEC	80%	n.a.	Positive	Negative	n.a.	Positive	Positive	Negative	Negative	n.a.
*X*	G2	−20%	Positive	Positive	n.a.	n.a.	Positive	n.a.	Negative	Negative	n.a.
*XI*	G2	−15%	Positive	Positive	Positive	n.a.	Positive	Negative	Negative	Negative	Positive
*XII*	G2	−15%	Weak positive	Positive	n.a.	n.a.	n.a.	Negative	Negative	n.a.	n.a.
*XIII*	G3	30%	Dot-like expression	Positive	Positive	n.a.	n.a.	Negative	Negative	Negative	n.a.
*XIV*	G1	<2%	Positive	Positive	Positive	n.a.	n.a.	n.a.	Positive	n.a.	n.a.
*XV*	G2	10%	Weak positive	Positive	n.a.	n.a.	n.a.	n.a.	n.a.	Negative	n.a.
*XVI*	G2	10%	Dot-like expression	Positive	n.a.	n.a.	n.a.	Negative	n.a.	n.a.	n.a.
*XVII*	G2	5%	n.a.	Positive	n.a.	n.a.	n.a.	n.a.	n.a.	n.a.	n.a.

n.a., not assessed.

Thyroid transcription factor 1 (TTF-1), a marker for metastases in NETs of pulmonary origin, and CDX2, a marker for metastases of gastrointestinal origin, were mostly negative in presacral neuroendocrine tumors. Only the presacral NEC was immunohistochemically positive for TTF-1, and one case of presacral NET was positive for CDX-2. Exclusion of a gastrointestinal primary tumor was performed by abdominal CT, gastroscopy, and colonoscopy in this case.

Vimentin—a marker for soft tissue tumors, but also expressed in various epithelial cancers—was examined immunohistochemically in only five presacral NETs, but it was detected in four of five cases.

Four presacral NETs were examined for PSAP by immunohistochemistry, and all resulted in positive detection of PSAP.

**Figure 2:**
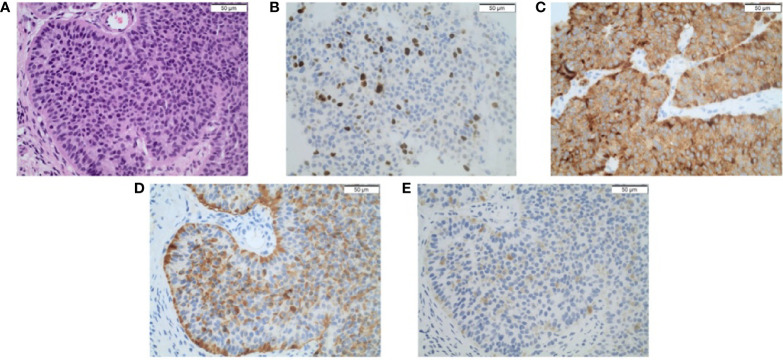
**(A)** Primary presacral neuroendocrine neoplasm stained using H&E (100×). Immunohistochemical staining (100×) shows a well-differentiated neuroendocrine neoplasm with a **(B)** Ki67 index of 7% and positivity for **(C)** synaptophysin, **(D)** chromogranin a, and **(E)** PSAP. Scale bars represent 50 µm.

A representative example of microscopic tumor morphology and immunohistochemical stainings is shown in **Figures 2A–E**. **Table 2** summarizes the results of the histopathological reports.

Cytokeratin 7 (CK7) was not detected by immunohistochemistry (n = 9). Cytokeratin 18 (CK18) was positive (n = 3). CD56, a non-specific marker for neuroendocrine tumors, was positive in four tumors and negative in one.

### Molecular Characterization

**Figure 3:**
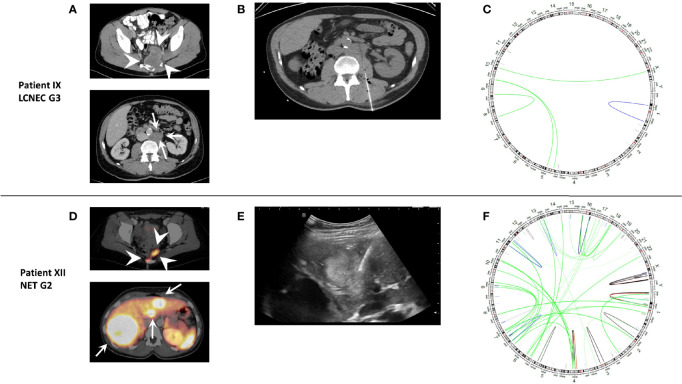
Representative imaging and genomic rearrangements of molecularly characterized patients IX (LCNEC G3) and XII (NET G2). **(A)** CT and **(D)** DOTATOC-PET/CT of presacral primary (white arrowheads) and metastases (white arrows). **(B)** CT-guided biopsy of retroperitoneal lymph node metastasis and **(E)** ultrasound-guided biopsy of liver metastasis for fresh tissue for genomic analysis. **(C, F)** Circle plots of genomic rearrangements. Despite slightly lower tumor mutational burden, case XII shows a much higher number of rearrangements as a sign of homologous DNA repair deficiency possibly due to a pathogenic frameshift SETD2 mutation.

Molecular diagnostics was performed in three patients to identify molecular targets: in one NET G3 (case XIII), a colorectal panel was applied, whereas one NEC G3 (case IX) and one NET G2 (case XII) were enrolled in the MASTER Trial and underwent whole-exome and whole-genome sequencing, respectively (**Figure 3**). All patients were microsatellite stable. In case XIII, no targetable alterations were detected, and the absence of a pathogenic TP53 mutation confirmed the diagnosis of NET G3. In the NEC G3 case, the tumor mutational burden (TMB) was intermediate with 4.32 non-coding mutations per megabase; besides a TP53 mutation, several cyclin pathway alterations (CDKN2A mutation, CCND1 mutation, CDK6 amplification) were detected. Case XII case showed a SETD2 frameshift insertion with presumably consecutive homologous DNA repair deficiency (HRD). TMB was low with 1.30 mutations per megabase.

### Circulating Biomarkers

The general circulating neuroendocrine biomarkers chromogranin A (CgA) were determined in 15 of 17 patients. CgA was only slightly elevated (n = 7) or normal (n = 8) at initial diagnosis. Tumor progression during follow-up was not accompanied by increasing CgA levels.

Serotonin—the marker hormone of the carcinoid syndrome—was determined in seven of our patients with presacral NETs; there was no elevation of serotonin in serum.

Neuron-specific enolase (NSE) is a marker for neuronal tissue, neuroendocrine cells, and in particular a circulating marker for poorly differentiated NEN. NSE serum levels were elevated in 6 of 10 patients, with only two patients showing a pronounced elevation >100 µg/L.

### Imaging

Imaging often reveals a solid tumor with sometimes cystic portions in the presacral space (see **Figure 1**). The morphological features of presacral NEN in CT and MR scans were unspecific.

SSTR imaging with specific PET-CTs like 68Ga-DOTATOC-PET/CT or SSTR scintigraphy was performed in 15/17 patients to exclude other potential primaries and for disease staging and treatment planning. Only one tumor showed no detectable SSTR expression. Most presacral NENs showed homogeneous SSTR expression (see for example **Figure 3D**), and only two tumors showed heterogeneous expression.

FDG-PET/CT was used in two patients with presacral NET and was not suitable for the detection of the primary tumor.

### Treatment in Patients With Presacral NEN

Curative treatment of presacral NENs is only possible in a locally limited stage, when surgical resection of the primary tumor represents the only chance of cure. In our series, most patients with presacral NENs were already in an advanced metastatic stage of disease at the time of diagnosis. Therefore, a palliative systemic therapy was initiated in most cases.

However, even in an already metastatic stage, surgical resection of the primary tumor may be considered for the treatment of symptoms due to the mass effect of the primary tumor. In our cohort, the primary tumor was resected in nine cases, in four patients with localized disease, in two patients with metastatic disease in curative intent (combined with resection of metastases), and in three metastatic patients in palliative intention.

In total, 14 patients with presacral NENs received therapy with SSA. Half of the patients showed stable disease at least until the first follow-up. The other patients underwent therapy escalation due to progression (n = 6) at first follow-up or intolerance (n = 1). During the course of disease, patients frequently showed progression of presacral NEN under SSA; therefore, treatment with SSA was usually not sufficient for growth control in the long term.

In our study population, three patients received treatment with everolimus and did not benefit from this therapy due to progression in the first follow-up. One patient received the tyrosine kinase inhibitor cabozantinib as the seventh line of therapy and showed stable disease [progression-free survival (PFS) >7 months]. The dose of cabozantinib was reduced due to side effects, but treatment was continued at last follow-up.

With good SSTR expression in almost all presacral NEN, PRRT was initiated 15 times in a total of 12 patients. Two patients developed a complete remission, four patients a partial remission, and five patients a stable disease. Two patients developed a mixed response and one a progressive disease. The result of one performed PRRT could not be assessed due to pending staging. Overall, the 14 PRRT responses assessed led to a clinical benefit in 11 cases, giving a clinical benefit rate of 79%.

Liver-directed therapies also proved to be a useful therapeutic approach. One patient developed partial remission after SIRT. Two patients received a transcatheter arterial chemoembolization (TACE) and showed partial remission or stable disease.

Presacral NENs showed limited sensitivity to cytotoxic agents. Overall, presacral NENs responded poorly to chemotherapy. Seven patients received palliative chemotherapy (4× platinum-based chemotherapy with etoposide, 2× temozolomide/capecitabine, and 1× paclitaxel +/− carboplatin). Only the patient with LCNEC showed partial remission under chemotherapy (cisplatin/etoposide). All other patients did not benefit from chemotherapy.

Radiotherapy of the primary tumor was performed in four patients by external beam radiation therapy and in one patient as particle therapy. Three patients developed stable disease and two partial remission after radiotherapy.

### Prognosis

Nine patients are currently still alive and in follow-up. Two patients were lost to follow-up. During a median follow-up time of 44.5 months (mean, 56.0 months; range, 0.73–212.4 months), six patients died 0.7–56.2 months after diagnosis of presacral NEN. Three of these patients died of their presacral NEN. The other three had an unknown cause of death.

**Figure 4:**
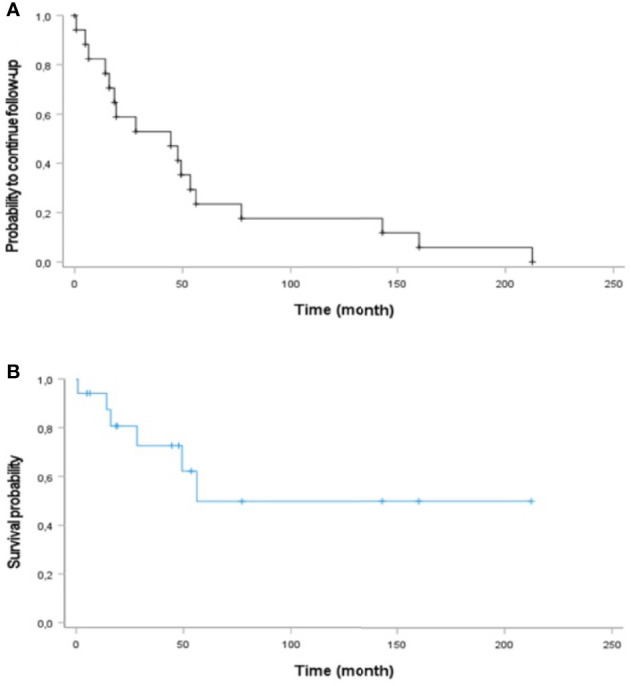
Kaplan–Meier curve analysis of **(A)** duration of observation and **(B)** overall survival of patients with primary presacral NEN (n = 17).

The Kaplan–Meier plot of duration of observation and overall survival is shown in **Figure 4**.

## Discussion

To the best of our knowledge, this is the largest study of presacral NEN so far. Besides clinical and pathological characteristics, we extensively analyzed the efficacy of different systemic therapeutics.

The pathological characteristics are in line with previous reports on presacral NENs. Expression of the neuroendocrine markers chromogranin A, synaptophysin, and CD56 is common, with CgA staining often being only weakly positive or even negative in some cases. When stained, Ck7, CDX2 (a marker of gastrointestinal origin), and TTF1 (a marker of pulmonary origin in NETs) were mostly negative, whereas PSAP, Ck18, and vimentin were mostly positive. This immunohistochemical profile resembles the profile of rectal NEN (11), which seems quite reasonable considering the hypothesis of a common ontogenetic origin from the embryonal hindgut (34). The similarity to rectal NEN is also supported by the only reported case of molecular profiling in presacral NEN we are aware of (19): here, an intestinal L cell was suggested as a putative cell of origin, with L-cell phenotype being reported in about 80% of rectal NETs (77). Most patients in our study showed well differentiated morphology with the vast majority being classified as NET G2. High-grade histology was detected in four patients, well-differentiated NET G3 in three, and poorly differentiated neuroendocrine carcinoma in one. This NEC was also the only TTF1-positive case in our cohort, demonstrating that TTF1 positivity is commonly observed in NEC of different origins and not a marker of pulmonary primary for NEC (in contrast to NET) (78).

Clinically, many patients showed local symptoms like pain and impairment of defecation as local symptoms of the tumor. However, in a remarkable proportion of patients, presacral NEN was identified as primary tumor of diagnosed NEN metastasis, and patients were referred to the centers as CUP NET cases.

Up to date, most cases of presacral NENs are published as single case reports or small series (14–72) (summarized in **Table 3**). In most of those cases, presacral NENs are treated locally with resection, and there is limited information on follow-up, metastasis, and systemic treatment. The largest case series that we could identify included 10 patients and reported on outcomes of different systemic therapeutic strategies for advanced disease (72). In our cohort, local resection was performed in 8 of 17 patients, half of them in palliative intention to treat local complaints. Several cases were treated with percutaneous radiotherapy, with encouraging results regarding local control and symptomatic improvement. In the literature, an association of presacral NENs with tailgut cysts and teratomas has been described. Additionally, an association with Currarino syndrome can be observed, an autosomal-dominant disorder caused by mutations in the motor neuron and pancreas homeobox 1 (MNX1) gene and characterized by presacral mass, sacral dysgenesis, anorectal anomalies (21, 49, 53). Most remarkably, whereas tailgut cysts or at least partially cystic primary tumors were observed in some of our patients, only one association with teratoma and none of other abnormalities like anorectal malformations or Currarino syndrome were present. Those abnormalities are quite often described in the case reports cited above; in the other larger case series of n = 10, only one patient presented with a teratoma. The observed difference between case reports and case series (including our analysis) could be attributed to a publication bias of more spectacular histological constellation for case reports and referral bias to centers where patients with advanced metastatic are more likely to be referred to. In our cohort, 12 of 17 patients presented with distant metastasis at first diagnosis, whereas more than 80% of the cases reported in the literature were localized or locally advanced.

**Table 3:** Previously reported cases of presacral NEN.

Reference	Age	Sex	Histology	Anomalies	IHC +: positive (+): weak or focal positive −: negative	Metastases synchronous	Metastases metachronous	Treatment (best response, PFS in months)	Follow-up (months) ^†^deceased
(42) Fiandaca 1988	35	F	NET	Teratoma	NR	LYM, HEP, OTH (ovary)		Surgery, PEB (NR)	NR (12 presurgery)
(70) Noshiro 1990	48	F	NET	–	NR	LYM		Surgery (CR, 24 og)	24
(60) Addis 1991	57	F	NET	–	CAM5.2+, S100(+), NSE(+), Vimentin-, GFAP-			Surgery (NR, 12 og)	12
(64) Lin 1992	18	F	NET	Tailgut cyst	NR			NR	NR
(55) Edelstein 1996	51	F	NET	–	NR			Surgery (CR, 18 og)	18
(71) Horenstein 1998	19	F	NET (Ki67 NR)	Tailgut cyst	CgA+, Syn+, NSE+, Cam5.2+, S100−, GFAP−			Surgery (CR, 48 og)	48
(71) Horenstein 1998	19	F	NET (Ki67 NR)	–	CgA+, Syn+, NSE+, Cam5.2+, GFAP−, NeuF−, serotonin−, Somatostatin−, VIP−, Gastrin−, Calcitonin−			2x Cis/Eto/Ifo, 3x Doxo/DTIC/Cyclo, embolization (SD, 12 og) Surgery (CR, 36 og)	48
(71) Horenstein 1998	21	F	NET (Ki67 NR)	–	CgA+, Syn+, NSE+, Cam5.2+, Ck7−, Ck20−, GFAP−, serotonin−, somatostatin−, VIP−, Gastrin−, Calcitonin−		LR, OTH (Breast)	Surgery (CR, 12) Surgery Breast (CR, 1)	13
(68) Gorski 1999	42	F			NR			NR	
(56) Oyama 2000	52	M	NET G1 (Ki67 NR)	Tailgut cyst	NR			Surgery (CR, 6 og)	6
(43) Prasad 2000	69	F	NEC (Ki67 NR)	Tailgut cyst	Ck+, CgA+			Surgery(CR, 24og)	24
(58) Theunissen 2001	51	F	NET G2 (“LCNEC”, Ki67 NR)	–	MNF116+, Vimentin(+), CEA−, CA125−, S100−, CgA+, Syn+			Cis/Eto (SD, 3)	3 †
(31) Mourra 2003	68	M	NET/NEC (Ki67 NR)	Tailgut cyst	NSE+, CgA+, Syn+, Ck+, EMA+, PSA−, CD45−			Surgery (CR, 12 og)	12
(26) Jacob 2004	42	F	NR	Tailgut cyst	NR			Surgery (NR)	NR
(16) Song 2004	41	F	NET (Ki67 NR)	Tailgut cyst	AE1/3+, Syn+, CgA+		HEP, BRA	Surgery (CR, 12) 5-FU (NR, 3) RT BRA (NR)	15
(48) Urioste 2004	22	M	NR	Teratoma, Currarino	NR			NR	
(18) Luong 2005	37	M	NET G1 (Ki67 2.9%)	Teratoma	Ck+, Syn+, NSE+, CgA−,	HEP, LYM, OSS		Lan (PD, 10) Lan (NR, 3)	18
(28) Mathieu 2005	49	F	NET (Ki67 NR)	Tailgut cyst	NR			Surgery (CR, 24 og)	24
(34) Kim 2007	58	F	NET (Ki67 NR	Imperforate anus	Syn+, CgA+, NSE+, Ck+, S100−			Surgery (CR, 10 og)	10
(24) Liang 2008	51	F	NET G2? (Ki67 > 1%)	Tailgut cyst	ER+, PR(+), Syn+, CgA+, PanCk+,			Surgery (NR)	NR (3 presurgery)
(20) La Rosa 2010	73	F	NET G1 (Ki67 < 2%)	Tailgut cyst	Syn+, CgB+, VMAT2+, SSTR2A+, PAP+, Ghrelin+, CgA+, Serotonin+, Somatostatin+, Ck20+, CDX2−, VMAT1−, PP−, YY−, GRP−, Gastrin−, glicentin−, encephalin−, GFAP−, ER−, PR−, AR−, Ck7−, TTF1−			Surgery (CR, 5)	5 (36 presurgery)
(65) Pendlimari 2010 (32) Liu 2020	22	F	NET G2 (Ki67 5%)	Currarino, teratoma	CgA+, Syn+, CD56(+)	LYM		Surgery (CR, 24 og)	24
(59) Ciotti 2011	44	F	NET G1/G2 (Ki67 < 10%)	Currrarino, teratoma	CgA+, Syn+	LYM	LR, LYM, HEP	Surgery (CR, 16) Surgery LR, LYM (CR, 24) Octreotide, CT (PD)	40 †
(69) Harbeck 2011	39	F	NET G2 (Ki67 5%)/NEC G3 (Ki67 30%)	–	CgA+, Syn+, Ck+, SSTR2+, serotonin−, glucagon−, somatostatin−		LYM, OSS	Surgery (CR, 30) PRRT (PR, 6 og)	36
(30) Spada 2011	63	F	NET G1 (Ki67 < 2%)	Tailgut cyst	AE1/3+, Syn+, PP+, AP+, CgA(+)	HEP		Surgery (CR, 25 og)	25
(30) Spada 2011	41	F	NET G2 (Ki67 18%)	Tailgut cyst	CgA+, Syn+, AE1/3+, SSTR2+	HEP	PLE, OSS, OTH (ovary)	Surgery (PR, NR) Carbo/Eto for HEP (NR, 7) PRRT (PR, NR) Surgery for PLE+Ovary (NR, NR) SSA (SD, NR) PRRT (SD, NR)	79
(62) Wöhlke 2011	55	F	NET G2 (Ki67 20%)	Tailgut cyst	AE1/3+, Syn+, CgA(+), somatostatin+, glucagon−, insulin−, gastrin−, CDX2−	LYM, HEP	OSS	Surgery, PRRT (CR, 22) PRRT (PR, 8)	30
(66) Zhong 2012	48	F	NET G1 (Ki67 1%)	–		OTH (muscle)		Surgery, cis/eto/doxo/cyclo RT (SD, 36 og)	36
(63) Zoccali 2012	64	M	NEN (Ki67 NR)	Tailgut cyst	AE1/3+, Syn+, CgA−, p63−			Surgery (NR)	NR
(57) Damato 2013	24	F	NET (Ki67 NR 5%)?	Tailgut cyst	Vimentin+, Ck+, S100−, Syn+, PSAP+			Surgery (CR, 3 og)	3
(41) Misawa 2013	53	F	NET/NEC (Ki67 20–60/70%)		AE1/3+, CAM5.1+/−, KL1+/−, S100 +/−, NSE+, Ubiquitin+, CD56+, CgA−, Syn+, LCA−, SMA−, Desmin−, CD10−/+, CD34−, HMB45−, GCDFP15−		LR, PER, PUL	Surgery (NR, 4) RTX for LR (NR, 4)	11 †
(67) Simpson 2014	64	F	NEN (Ki67 NR)	Teratoma	NR	NR		Surgery (NR)	NR
(39) Abukar 2014	61	M	NET G2 (Ki67 low)	Tailgut cyst	PAP+, CD56(+), Syn+, CgA+, MNF116+, AE1/3+			Surgery (NR)	NR
(17) Charalampakis 2014	35	M	NET G1 (Ki67 < 1%)?	Tailgut cyst	PanCk+, CgA+, Syn+			Surgery (CR, 36 og)	36
(37) Kim 2014	49	M	NET G2 (Ki67 5%)	Tailgut cyst	CgA+, Syn+, CD56+			Surgery, w&w for residual tumor (SD, 14 og)	24
(61) Menter 2014	69	M	NET G2 (Ki67 10%), PLE: Ki67 15%	–	PSAP+, TTF1−, PSA−, Ck20(+), CD56+, CgA+, Syn+, SSTR2+, Ck22+, Ck7−, EMA−, ERG−, S100−,	OSS, HEP, PUL, LYM, OTH, heart, duodenum, mesenterium		Surgery, RT (PR, 36)	72^†^
(22) Mitsuyama 2015	53	M	NET G2 (Ki67 12.5%)	Tailgut cyst	Vimentin+, panCk(+), EMA−, S100−, CD99−, CgA+, Syn+, SSTR2+			Surgery (CR, 10) Octreotide (NR) Irradiation (NR, 10) Everolimus (NR, 8 og)	28
(52) Sable 2014	35	F	NET G1 (Ki67 2%)	Teratoma	Ck+, Syn+			Surgery, CT	NR
(35) Falkmer 2015	57	M	NET G2 (Ki67 5–10%)	–	AE1/3+, Syn+, CgA+, CgB+, Ghrelin+, PYY(+), Motilin(+), VMAT2−, Serotonin−, Gastrin−, GIP−, CGRP−, CART−, Calcitonin−, ACTH−, Secretin−, VIP−, NRK−, Insulin−, IAPP−, glucagon−, GLP1−, GRP−, neurotensin−	LYM, OSS	LR, SKI, BRA, OTH (soft tissue, kidney, heart)	Watch&wait (NR, 27) Surgery (8) RTX (4) STZ/5-FU (PD, NR) PRRT (NR,19) Octreotide (NR, NR) Octreotide-HD (NR, NR) RTX (NR, 13) PRRT (NR, 25) PRRT (PR, 9)	135 †
(38) Jehangir 2016	74	M	NET (Ki67 NR)	Tailgut cyst	Syn+, NSE+, CgA−			Surgery (CR, 60 og)	60
(40) Ferrer-Márquez 2017	NR	NR	NET (Ki67 NR)	NR	NR			Surgery (CR, 24 og)	24
(40) Ferrer-Márquez 2017	NR	NR	NET (Ki67 NR)	NR				Surgery (CR, 24 og)	24
(40) Ferrer-Márquez 2017	NR	NR	NET (Ki67 NR)	NR				Surgery (CR, 6 og)	6
(45) Mora-Guzmán 2017	56	F	NET G1 (Ki67 < 2%)	Tailgut cyst	AE1/3+, CD56+, Syn+, CgA+			Surgery (CR)	7
(29) Al Khaldi 2018	53	F	NET G2 (Ki67 5-10%)	Tailgut cyst	CgA+, Syn+, Cam5.2+, AE1/3(+), CD56(+)		LYM, PUL, HEP	Surgery (CR, 24) SSA for LYM+PUL (NR, 6) SZT/5-FU (SD, 12) Everolimus (PD, 4)	46
(19) Erdrich 2018	77	M	NET G2 (Ki67 8.6%) HEP: NET G2 (Ki67 6.4%)	Tailgut cyst	CgA(+), Syn+	HEP		Surgery primary + HEP	NR
(14) Iwata 2019	25	F	NET G2, (Ki67 20%)	Tailgut cyst	Ck+, Syn+, CgA+, ER−, PR−			Surgery (NR)	NR (8 presurgery)
(51) Soyer 2018	14	M		Tailgut cyst					NR
(72) Yang 2018	39	F	NET G2 (Ki67 5–10%)	Tailgut cyst	CgA+, Syn+, Serotonin−, TTF1−, CDX2−, PAX8−, PP−		LR, HEP, PUL, PER, OTH (ovaries)	Surgery (CR, 12) Surgery + SSA for LR (CR,12) Surgery LR + PER, ablation (NR, 41) Surgery + RT for LR + PER + OTH (NR, 36) SIRT + SSA (NR, NR)	120
(72) Yang 2018	41	F	NET (Ki67 NR)	Teratoma	Syn+, CgA+, Ck+, AE1/3+, S100+		OTH (ovaries, retroperitoneal)	Surgery, SSA (CR, 48) Surgery (ovaries, retroperitoneal) (NR, NR)	48
(72) Yang 2018	45	F	NET (Ki67 NR), BRA: NET G2, Ki67 18%	Anterior sacral meningocele, tailgut cyst	CAM5.2+, CgA+, Syn+, CDX2+		BRA, LYM, HEP, OSS	Surgery (CR, 9) Surgery brain (CR, 3) Octreotide, PRRT LR, LYM, HEP (SD, 36) PRRT for HEP, OSS (PR, NR)	46 (156 presurgery
(72) Yang 2018	46	M	NET G2 (Ki67 15%) BRA: NET G2 (Ki67 12%)	Tailgut cyst	Syn+, CgA+, AE1/3+, TTF1−, CDX2−	HEP, OSS		FOLFOX + Bev (SD?, 14 og) Everolimus + Oct, Irradiation (NR, 12) Surgery (BRA)	28
(72) Yang 2018	75	M	NET G1, Ki67 < 1%	–		LYM, OSS, PUL	HEP	Surgery, RTX, Oct (NR, 47) SIRT, Oct (PR, 27 og)	78
(72) Yang 2018	42	F	NET G2 (Ki67 6%)	–	Syn+, CD56+, NSE+, WT1+,		HEP	Surgery (CR, 12) Oct (NR, 25) Oct + CC-223 (NR, 9) SIRT (PR, 11) Surgery pancreas (NR, NR) Tem (PD, NR) Sunitinib (NR, NR) PRRT (SD, NR)	68
(72) Yang 2018	41	F	NET G2 (Ki67 13%)	–		PUL, HEP, OSS	BRA	Lan + IFN (NR, 9) Pazopanib (NR, 16) RT OSS BRA (SD, 6 og)	36
(72) Yang 2018	44	F	NEC G3 (Ki67 80–90%)	–	Ck+, Syn+, CD56+	LYM		Cis/Eto, RTX (PR, 5 og)	5
(72) Yang 2018	77	F	Large-cell NET/NEC (Ki67 50%)	–	Syn+, CD56+, Villin+			Carbo/Eto (SD, 3 og) Oct (PD, 4)	7
(72) Yang 2018	50	F	NET (Ki67 NR)	–		LYM, HEP, OTH (pancreas)		Tem/Cap (NR), Oct (NR)	NR
(21) Chatani 2019	59	F	NET G2 (Ki67 NR), Adenocarcinoma	Teratoma, Currarino	Syn+, CgA+			Resection, RT (CR, 8 og)	8
(49) Coetzee 2019	60	F	NET G2 (Ki67 14%)	Teratoma, Currarino	CgA+, Syn+			Surgery (CR, 18 og)	18
(53) Colombo 2019	46	M	NET (Ki67 NR)	Currarino, dermoid cyst	NR				NR
(36) Kim 2019	78	M	NET G2 (Ki67 6.6%)	–	CgA+, Syn+, CD56+, Ck7-, TTF1-, CDX2-	HEP	PER	Surgery HEP (CR, 48) Octreotide + everolimus (SD, 14) Pazopanib (NR, 14) PRRT (SD, 2 og)	96
(15) Lee 2019	33	F	NET G1 (Ki67 1-2%)	Tailgut cyst	AE1/3+, Syn+, CgA+, CDX2(+), ER(+), Ck7(+), Ck20-			Surgery (NR, NR)	NR (96 presurgery)
(46) Olczak 2019	49	M							
(50) Rod 2019	51	M	NET (Ki67 NR)	Currarino	NR			Surgery (NR, NR)	60
(27) Sakr 2019		M	NET (Ki67 NR)	Tailgut cyst	NR			Surgery (NR, NR), RT (NR, NR)	NR
(33) Singh 2019	63	M	NET G1 (Ki67 < 1%)	Tailgut cyst	Syn+, Ck+, CgA−, GFAP−, SMHC−, p63−, CD56−			Surgery (NR, NR)	NR
(25) Zhang 2019	36	F	NET G3 (Ki67 30%)	–	Syn+, CD56+, Ck+			Surgery (CR, 1 og)	1
(23) Kodera 2020	68	F	NET G1 (Ki67 < 2%)	Tailgut cyst	CD56+, SSTR2A+, PP+, PR(+), CgA−, p53−, ER−, gastrin−, serotonin−, somatostatin−, CDX2−, TTF1−			Surgery (CR, 12 og)	12
(32) Liu 2020	75	F	NET G2 (Ki67 3%), HEP: NET G2 (Ki67 6.8%)	Tailgut cyst, Currarino	NR	HEP		Octreotide (PD, 11) PRRT (NR, NR)	11
(32) Liu 2020 (54) Scott 2021	35	F	NET G2 (Ki67 4%)	Currarino, tailgut cyst	Syn+, SSTR2A+, Islet-1+, CgA−, Ck20−, TTF1−, CDX2−, PAX8−, GATA3−, Inhibin−Desmin−, S100−	LYM, OSS		Surgery, Octreotide (SD, 22 og) PRRT (NR, NR) Oct high dose (SD, NR)	36
(47) Rebelo 2020	48	M	NET G2 (Ki67 6%)	Tailgut cyst, Currarino	CD56+, Syn+, CgA−		HEP, OSS, OTH (spleen)	Surgery (CR, 18) CT	18
(54) Scott 2021	38	M	NET G2 (Ki67 7.5%), LYM: NET G2 (Ki67 9%)	Currarino, tailgut cyst	Syn+, CgA+, SSTR2A+, Islet−1+, PAX6+, CDX2−, TTF1−		LYM	Surgery (CR, 24) Surgery LYM (CR, NR)	24
(54) Scott 2021	62	F	NET G1 (Ki67 < 1%)	Currarino, teratoma	AE1/3+, CAM 5.2+, Syn+, CD56(+), Ck5/6−, p63−, S100−, desmin−, CD34−, CD45−, CgA−			Surgery (CR, 12 og)	12

Histopathology was adapted to the most current WHO 2019 classification according to the description in the report.

AP, acid phosphatase; AR, androgen receptor; BRA, brain; CAM, cell adhesion molecule; Cap, capecitabine; Cis, cisplatin; Ck, cytokeratin; CT, chemotherapy; CgA, chromogranin A; CgB, chromogranin B; CR, complete remission; cyclo, cyclophosphamide; doxo, doxorubicin; DTIC, dacarbazin; EMA, epithelial membrane antigen, ER, estrogen receptor; eto, etoposide; F, female, GCDFP, gross cystic disease fluid protein; GFAP, glial fibrillary acidic protein; GIP, gastric inhibitory peptide; GRP, gastrin releasing peptide; HEP, liver; Lan, lanreotide, LR, local recurrence; LYM, lymph nodes; M, male; NF, neurofilament; NR, not reported; NSE, neuron-specific enolase; Oct, octreotide; og, ongoing; OSS, bone; OTH, other, PD, progressive disease; PER, peritoneum; PgR, progesterone receptor; PLE, pleura; PP, pancreatic polypeptide; PR, partial remission; PRRT, peptide receptor radionuclide therapy; PSAP, prostatic specific acidic phosphatase; PUL, lung; PYY, peptide YY; RT, radiotherapy; SD, stable disease; SKI, skin; SSA, somatostatin analogue; SSTR, somatostatin receptor; syn, synaptophysin; Tem, temozolomide; VIP, vasoactive intestinal peptide; VMAT, vesicular monoamine transporter; w&w: watch & wait.

When taking the systemic treatments applied for presacral NEN in our analysis into context, beside comparing them to NEN in general, a special focus should be laid on rectal NEN regarding their resemblance to presacral NEN as discussed above.

SSAs are among the first approved drugs for disease stabilizations for well-differentiated NETs, with octreotide for midgut NET in the PROMID trial (79) and lanreotide for enteropancreatic NET in the CLARINET trial (80). However, the use of SSAs in rectal NET is up for debate, since lanreotide failed to show PFS benefit *vs.* placebo in the CLARINET trial with very few rectal NET patients included. Nevertheless, SSAs are also commonly reported as effective treatment for presacral NET, with octreotide in 13 cases (22, 32, 36, 72), lanreotide in 1 case (72), and an unspecified SSA in 4 cases (29, 72). Fifteen patients in our analysis received SSAs, half of them lanreotide. Disease stabilization was observed in 50% (7/14).

PRRT is an effective treatment for NET of various locations since the mid-90s. With the conclusion of the NETTER trial, PRRT has shown its efficacy in a randomized phase III setting for intestinal NET (81), gaining approval in many countries. In rectal NET, PRRT has shown efficacy in case series (82). PRRT is reported in nine cases with presacral NET, all with encouraging long-term stabilizations and remission (32, 35, 36, 72). In accordance to this, PRRT was also one of the most effective treatments observed in our cohort, resulting in 43% responses (6/14) and 36% disease stabilizations (5/14).

The mTOR inhibitor everolimus has been approved for extrapancreatic NET after the positive phase III RADIANT-4 trial (83). The subgroup of rectal NETs in this trial showed significant PFS prolongation in this trial. Four cases of treatment of presacral NET are reported in the literature (22, 36, 72), with one showing long-term disease control, two short-term stabilizations, and one progressive disease. In our analysis, all three patients receiving everolimus showed disease progression.

The multi-tyrosine kinase inhibitor cabozantinib has shown promising antitumor activity in a preliminary report of a phase II trial for pancreatic and extrapancreatic NETs (72). We report the first patient receiving cabozantinib for metastatic presacral NET with an encouraging long-lasting disease stabilization in a heavily pretreated patient.

Cytotoxic chemotherapy is the main systemic treatment modality for NEN G3 and an effective option for pancreatic NET G1/G2 (73). In presacral NET, applications of the protocols FOLFOX (n = 1) (72), temozolomide + capecitabine (n = 2) (72), cisplatin + etoposide (n = 4) (58, 72), and carboplatin-etoposide (n = 2) (72) were reported, with heterogenous results. In our analysis, chemotherapy was applied seven times to six patients, mostly with unfavorable response. While well-differentiated presacral NET did not sufficiently respond to chemotherapy, treatment of the presacral neuroendocrine carcinoma with cisplatin + etoposide resulted in a partial remission.

Of three patients receiving molecular diagnostics, potential targetable alterations were detected in two patients: alterations in the cyclin pathway as potential target for a CDK inhibitor and HRD as a potential target for PARP inhibition and platinum-based chemotherapy most probable due to a SETD2 frameshift insertion (84–86). This is remarkable, since the only other case of molecularly profiled presacral NET reported earlier (19) showed a BRCA1 mutation, which also commonly leads to HRD.

Our study has several limitations, mainly due to its retrospective nature. On the other hand, considering the rarity of the disease, a prospective or even randomized trial is most likely not feasible. Furthermore, a central pathological or radiological review was not performed. However, all patients were included by experienced high volume NEN centers with well-established multidisciplinary diagnostic and therapeutic pathways.

In conclusion, we report the largest analysis of clinicopathological characteristics and treatment outcomes for presacral NEN so far. Presacral NENs are usually non-functioning and primarily cause locoregional symptoms. Plasma CgA levels are usually not elevated. Presacral NEN should be considered as possible primary in CUP-NET, especially when the immunohistochemical profile resembles a hindgut NET, and a rectal primary is excluded endoscopically. Functional imaging with SSR-based PET-CT is helpful for primary tumor identification and treatment planning. Local control could be achieved *via* radiotherapy. SSAs demonstrated limited efficacy, whereas PRRT showed promising activity for advanced disease. In our cohort, everolimus and chemotherapy were largely ineffective. Molecular diagnostics showed potential targetable alterations in selected cases. Further prospective evaluation and molecular characterization of this rare tumor entity are needed.

## Data Availability Statement

The data analyzed in this study is subject to the following licenses/restrictions: The dataset supporting the conclusions of this article is available on request by contacting the authors. **Tables 1**, **2** and **Figure 3** build the dataset. Requests to access these datasets should be directed to sprengea@uni-marburg.

## Ethics Statement

The study was conducted in accordance with the Declaration of Helsinki. All patients were included in the local disease databases conducted with approval of the local ethics committees at the respective sites. Written informed patient consent and approval for data collection and analysis were obtained upon admission to our institutions. For the use of the images, an additional consent was obtained in the selected cases.

## Author Contributions

ARi and DL contributed to conception and design of the study. SM, LA, JS, HL, SKru, ARi, ZK, and AK carried out the acquisition of data. SM, LA, and ARi analyzed and interpreted the data and drafted the manuscript. SM performed statistical analysis. ARa performed immunohistochemical stainings and pathological review. DL contributed PET-CT images. SKre provided molecular data and participated in data interpretation. TG was involved in data interpretation and critical review of the manuscript draft. All authors contributed to the article and approved the submitted version.

## Conflict of Interest

ARi has received honoraria for presentations and advisory boards from AAA, Advanz Pharma, Falk, IPSEN and Novartis. LA has received honoraria and travel expenses from Ipsen and Novartis. JS has received honoraria for presentations and advisory boards from Advanz Pharma, IPSEN and Novartis, and research grants from Riemser Pharma and Novartis. HL reports personal fees and grants from Novartis, and personal fees from Ipsen and AAA, outside the submitted work. AK has received honoraria for presentations from Ipsen and Novartis. TMG has received funding from IPSEN, Pfizer, and Novartis for joined research projects, participation in advisory boards, and lectures.

The remaining authors declare that the research was conducted in the absence of any commercial or financial relationships that could be construed as a potential conflict of interest.

## Publisher’s Note

All claims expressed in this article are solely those of the authors and do not necessarily represent those of their affiliated organizations, or those of the publisher, the editors and the reviewers. Any product that may be evaluated in this article, or claim that may be made by its manufacturer, is not guaranteed or endorsed by the publisher.
